# Diacetonitrile[*N*,*N*′-bis(2,6-diisopropyl­phenyl)ethane-1,2-diimine]dichloridochromium(II) acetonitrile solvate

**DOI:** 10.1107/S1600536809040094

**Published:** 2009-10-10

**Authors:** Stephan Peitz, Normen Peulecke, Bernd H. Müller, Anke Spannenberg, Uwe Rosenthal

**Affiliations:** a Leibniz-Institut für Katalyse e. V. an der Universität Rostock, Albert-Einstein-Strasse 29a, 18059 Rostock, Germany

## Abstract

The title compound, [CrCl_2_(CH_3_CN)_2_(C_26_H_36_N_2_)]·CH_3_CN, was synthesized by the reaction of CrCl_2_(THF)_2_ with *N*,*N*′-bis­(2,6-diisopropyl­phen­yl)ethane-1,2-diimine in dichloro­methane/acetonitrile. The chromium center is coordinated by two N atoms of the chelating diimine ligand, two chloride ions in a *trans* configuration with respect to each other, and by two N atoms of two acetonitrile mol­ecules in a distorted octa­hedral geometry.

## Related literature

For derivatives of the title compound, see: Turki *et al.* (2006 ▶); Kreisel *et al.* (2007 ▶); Ghosh *et al.* (2008 ▶). For catalytic features of diimine and PNP ligands, see: tom Dieck & Kinzel (1979 ▶); Bart *et al.* (2004 ▶); Huang *et al.* (2007 ▶); Wöhl *et al.* (2009 ▶).
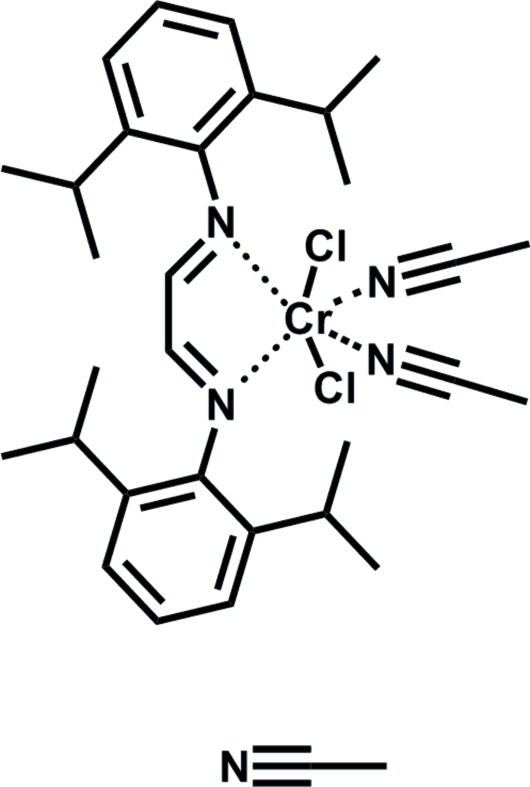


## Experimental

### 

#### Crystal data

[CrCl_2_(C_2_H_3_N)_2_(C_26_H_36_N_2_)]·C_2_H_3_N
*M* *_r_* = 622.63
Monoclinic, 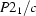

*a* = 18.7305 (6) Å
*b* = 13.2462 (5) Å
*c* = 13.9582 (4) Å
β = 97.838 (2)°
*V* = 3430.8 (2) Å^3^
*Z* = 4
Mo *K*α radiation
μ = 0.52 mm^−1^
*T* = 200 K
0.5 × 0.5 × 0.4 mm

#### Data collection

Stoe IPDSII diffractometer
Absorption correction: numerical (**X-SHAPE** and **X-RED32**; Stoe & Cie, 2005 ▶) *T*_min_ = 0.761, *T*_max_ = 0.847
53468 measured reflections
7489 independent reflections
6233 reflections with *I* > 2σ(*I*)
*R*_int_ = 0.036

#### Refinement

*R*[*F* ^2^ > 2σ(*F* ^2^)] = 0.043
*wR*(*F* ^2^) = 0.108
*S* = 1.20
7489 reflections
364 parameters
H-atom parameters constrained
Δρ_max_ = 0.47 e Å^−3^
Δρ_min_ = −0.40 e Å^−3^

### 

Data collection: *X-AREA* (Stoe & Cie, 2005 ▶); cell refinement: *X-AREA*; data reduction: *X-AREA*; program(s) used to solve structure: *SHELXS97* (Sheldrick, 2008 ▶); program(s) used to refine structure: *SHELXL97* (Sheldrick, 2008 ▶); molecular graphics: *XP* in *SHELXTL* (Sheldrick, 2008 ▶); software used to prepare material for publication: *SHELXTL*.

## Supplementary Material

Crystal structure: contains datablocks I, global. DOI: 10.1107/S1600536809040094/im2148sup1.cif

Structure factors: contains datablocks I. DOI: 10.1107/S1600536809040094/im2148Isup2.hkl

Additional supplementary materials:  crystallographic information; 3D view; checkCIF report

## Figures and Tables

**Fig. 1.**
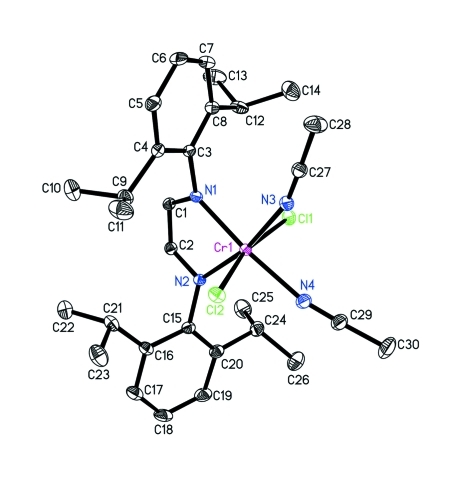
The molecular structure of the title compound showing the atom-labelling scheme. Displacement ellipsoids are drawn at the 30% probability level. Hydrogen atoms are omitted for clarity. The asymmetric unit additionally contains one solvent molecule acetonitrile which is not shown.

## supplementary crystallographic information

### Comment

1,2-Diiminoethane ligands, also called diazabutadiene ligands, have been used for many purposes in catalysis and coordination chemistry. Complexes with different metals including chromium have been investigated concerning their electronic and structural features (Turki *et al.*, 2006; Ghosh *et al.*, 2008). Dimerization of isoprene (tom Dieck *et al.*, 1979) and polymerization of ethene (Bart *et al.*, 2004; Huang *et al.*, 2007) are examples for catalytic investigations with this type of ligand. The shortest metal-metal bond at that time was observed in a dinuclear chromium complex with this ligand (Kreisel *et al.*, 2007).

We became interested in chromium complexes with this ligand during our studies on the selective oligomerization of ethylene *via* transition-metal catalyzed tri- or tetramerization, yielding 1-hexene or 1-octene (Wöhl *et al.*, 2009). Derived thereof we wanted to examine the *N*,*N*'-chelating ligand in combination with chromium in order to find differences and similarities in coordination and catalysis compared to other oligomerization systems. We deployed a simple preparation procedure that is described here, to obtain the complex for our screening experiments.

The molecular structure of the title compound shows that the chromium(II) center is coordinated by two N atoms of the chelating diazabutadiene ligand, (*i*-Pr)_2_C_6_H_3_–N═C(H)C(H)═N—C_6_H_3_(*i*-Pr)_2_, two chloride ions in *trans*-configuration with respect to each other and two acetonitrile molecules (Fig. 1). Its coordination geometry can be best described as distorted octahedral. Furthermore, the chelating ligand and the metal form a five-membered ring Cr(N═C–C═N), which is folded along the NN-axis by an angle of 162.5 (1)°. The asymmetric unit contains additionally one solvent molecule acetonitrile.

### Experimental

CrCl_2_(THF)_2_ (1.50 g, 5.66 mmol) and *N*,*N*'-bis(2,6-diisopropylphenyl)ethane-1,2-diimine (2.13 g, 5.66 mmol) were dissolved in 20 ml of dichloromethane at room temperature and stirred over night. After removal of all volatiles in vacuum the residue was washed with small amounts of *n*-hexane. Crystallization in acetonitrile yielded 0.59 g (27%) of brown single crystals suitable for X-ray analysis.

### Refinement

The H atoms were placed in idealized positions with d(C—H) = 0.98 (CH_3_) and 0.95–1.00 Å (CH) and refined using a riding model with *U*_iso_(H) fixed at 1.5 *U*_eq_(C) for CH_3_ and 1.2 *U*_eq_(C) for CH.

### Crystal data

**Table:**

[CrCl_2_(C_2_H_3_N)_2_(C_26_H_36_N_2_)]·C_2_H_3_N	*F*(000) = 1320
*M**_r_* = 622.63	*D*_x_ = 1.205 Mg m^−^^3^
Monoclinic, *P*2_1_/*c*	Mo *K*α radiation, λ = 0.71073 Å
Hall symbol: -P 2ybc	Cell parameters from 12659 reflections
*a* = 18.7305 (6) Å	θ = 2.1–29.6°
*b* = 13.2462 (5) Å	µ = 0.52 mm^−^^1^
*c* = 13.9582 (4) Å	*T* = 200 K
β = 97.838 (2)°	Prism, brown
*V* = 3430.8 (2) Å^3^	0.5 × 0.5 × 0.4 mm
*Z* = 4	

### Data collection

**Table:**

Stoe IPDSII diffractometer	7489 independent reflections
Radiation source: fine-focus sealed tube	6233 reflections with *I* > 2σ(*I*)
graphite	*R*_int_ = 0.036
ω scans	θ_max_ = 27.0°, θ_min_ = 1.9°
Absorption correction: numerical (*X-SHAPE* and *X-RED32*; Stoe & Cie, 2005)	*h* = −23→23
*T*_min_ = 0.761, *T*_max_ = 0.847	*k* = −16→16
53468 measured reflections	*l* = −17→17

### Refinement

**Table:**

Refinement on *F*^2^	Primary atom site location: structure-invariant direct methods
Least-squares matrix: full	Secondary atom site location: difference Fourier map
*R*[*F*^2^ > 2σ(*F*^2^)] = 0.043	Hydrogen site location: inferred from neighbouring sites
*wR*(*F*^2^) = 0.108	H-atom parameters constrained
*S* = 1.20	*w* = 1/[σ^2^(*F*_o_^2^) + (0.0286*P*)^2^ + 3.9005*P*] where *P* = (*F*_o_^2^ + 2*F*_c_^2^)/3
7489 reflections	(Δ/σ)_max_ = 0.001
364 parameters	Δρ_max_ = 0.47 e Å^−^^3^
0 restraints	Δρ_min_ = −0.40 e Å^−^^3^

### Special details

**Table:**

Geometry. All e.s.d.'s (except the e.s.d. in the dihedral angle between two l.s. planes) are estimated using the full covariance matrix. The cell e.s.d.'s are taken into account individually in the estimation of e.s.d.'s in distances, angles and torsion angles; correlations between e.s.d.'s in cell parameters are only used when they are defined by crystal symmetry. An approximate (isotropic) treatment of cell e.s.d.'s is used for estimating e.s.d.'s involving l.s. planes.
Refinement. Refinement of *F*^2^ against ALL reflections. The weighted *R*-factor *wR* and goodness of fit *S* are based on *F*^2^, conventional *R*-factors *R* are based on *F*, with *F* set to zero for negative *F*^2^. The threshold expression of *F*^2^ > σ(*F*^2^) is used only for calculating *R*-factors(gt) *etc*. and is not relevant to the choice of reflections for refinement. *R*-factors based on *F*^2^ are statistically about twice as large as those based on *F*, and *R*- factors based on ALL data will be even larger.

### Fractional atomic coordinates and isotropic or equivalent isotropic displacement parameters (Å^2^)

**Table:**

	*x*	*y*	*z*	*U*_iso_*/*U*_eq_	
N5	0.1588 (3)	0.3180 (4)	0.4602 (4)	0.1140 (18)	
C31	0.1283 (3)	0.3863 (4)	0.4315 (3)	0.0728 (13)	
C32	0.0890 (2)	0.4750 (3)	0.3954 (3)	0.0679 (11)	
H32A	0.1213	0.5336	0.4020	0.102*	
H32B	0.0490	0.4866	0.4325	0.102*	
H32C	0.0700	0.4652	0.3271	0.102*	
C1	0.27829 (12)	0.77519 (18)	0.13545 (17)	0.0227 (5)	
H1A	0.2894	0.8190	0.0857	0.027*	
C2	0.24076 (12)	0.68549 (18)	0.11524 (18)	0.0225 (5)	
H2A	0.2236	0.6656	0.0508	0.027*	
C3	0.32484 (13)	0.89587 (17)	0.25385 (17)	0.0215 (5)	
C4	0.27698 (13)	0.96797 (18)	0.28368 (18)	0.0249 (5)	
C5	0.30411 (14)	1.06144 (19)	0.3145 (2)	0.0301 (6)	
H5A	0.2729	1.1101	0.3365	0.036*	
C6	0.37644 (15)	1.0852 (2)	0.3138 (2)	0.0329 (6)	
H6A	0.3945	1.1490	0.3369	0.039*	
C7	0.42186 (14)	1.0162 (2)	0.2795 (2)	0.0315 (6)	
H7A	0.4705	1.0343	0.2762	0.038*	
C8	0.39764 (13)	0.91987 (19)	0.24949 (19)	0.0264 (5)	
C9	0.19649 (13)	0.9481 (2)	0.2747 (2)	0.0320 (6)	
H9A	0.1893	0.8732	0.2716	0.038*	
C10	0.16000 (18)	0.9923 (3)	0.1792 (3)	0.0591 (10)	
H10A	0.1831	0.9654	0.1257	0.089*	
H10B	0.1647	1.0660	0.1809	0.089*	
H10C	0.1088	0.9740	0.1699	0.089*	
C11	0.16168 (17)	0.9872 (3)	0.3605 (3)	0.0514 (9)	
H11A	0.1863	0.9579	0.4205	0.077*	
H11B	0.1107	0.9679	0.3524	0.077*	
H11C	0.1657	1.0609	0.3636	0.077*	
C12	0.44939 (14)	0.8477 (2)	0.2111 (2)	0.0335 (6)	
H12A	0.4234	0.7826	0.1952	0.040*	
C13	0.47405 (18)	0.8879 (3)	0.1183 (3)	0.0487 (8)	
H13A	0.4319	0.9012	0.0703	0.073*	
H13B	0.5049	0.8377	0.0927	0.073*	
H13C	0.5012	0.9507	0.1324	0.073*	
C14	0.51433 (16)	0.8257 (3)	0.2870 (3)	0.0483 (8)	
H14A	0.4977	0.7995	0.3457	0.073*	
H14B	0.5417	0.8880	0.3022	0.073*	
H14C	0.5453	0.7754	0.2616	0.073*	
C15	0.17107 (13)	0.55834 (18)	0.17315 (17)	0.0223 (5)	
C16	0.09994 (13)	0.5968 (2)	0.15552 (18)	0.0257 (5)	
C17	0.04407 (15)	0.5284 (2)	0.1314 (2)	0.0344 (6)	
H17A	−0.0040	0.5526	0.1185	0.041*	
C18	0.05677 (16)	0.4263 (2)	0.1257 (2)	0.0390 (7)	
H18A	0.0177	0.3812	0.1089	0.047*	
C19	0.12614 (16)	0.3896 (2)	0.1445 (2)	0.0374 (7)	
H19A	0.1342	0.3190	0.1413	0.045*	
C20	0.18483 (14)	0.45424 (19)	0.16800 (19)	0.0282 (5)	
C21	0.08221 (13)	0.7084 (2)	0.1614 (2)	0.0293 (6)	
H21A	0.1275	0.7449	0.1872	0.035*	
C22	0.05521 (16)	0.7513 (2)	0.0607 (2)	0.0401 (7)	
H22A	0.0913	0.7388	0.0174	0.060*	
H22B	0.0474	0.8242	0.0657	0.060*	
H22C	0.0098	0.7184	0.0348	0.060*	
C23	0.02642 (16)	0.7285 (3)	0.2299 (2)	0.0448 (8)	
H23A	0.0440	0.7012	0.2940	0.067*	
H23B	−0.0192	0.6957	0.2046	0.067*	
H23C	0.0188	0.8014	0.2348	0.067*	
C24	0.26105 (15)	0.4138 (2)	0.1798 (2)	0.0331 (6)	
H24A	0.2930	0.4647	0.2173	0.040*	
C25	0.28633 (18)	0.4018 (3)	0.0804 (3)	0.0496 (8)	
H25A	0.2807	0.4662	0.0456	0.074*	
H25B	0.2573	0.3499	0.0433	0.074*	
H25C	0.3371	0.3817	0.0889	0.074*	
C26	0.2691 (2)	0.3140 (2)	0.2351 (3)	0.0593 (10)	
H26A	0.2531	0.3227	0.2985	0.089*	
H26B	0.3198	0.2932	0.2437	0.089*	
H26C	0.2397	0.2622	0.1984	0.089*	
Cl1	0.39614 (3)	0.60550 (5)	0.26626 (5)	0.03066 (15)	
Cl2	0.19247 (3)	0.71492 (5)	0.38646 (4)	0.02861 (14)	
Cr1	0.29054 (2)	0.67426 (3)	0.31096 (3)	0.01897 (9)	
N1	0.29772 (10)	0.79602 (14)	0.22866 (14)	0.0200 (4)	
N2	0.23010 (10)	0.62865 (14)	0.19125 (14)	0.0191 (4)	
N3	0.35525 (11)	0.75068 (17)	0.42260 (15)	0.0273 (5)	
C27	0.38699 (14)	0.8105 (2)	0.46731 (19)	0.0298 (6)	
C28	0.42740 (19)	0.8893 (3)	0.5229 (2)	0.0474 (8)	
H28A	0.4790	0.8737	0.5293	0.071*	
H28B	0.4123	0.8932	0.5873	0.071*	
H28C	0.4183	0.9541	0.4898	0.071*	
N4	0.29488 (12)	0.54279 (17)	0.39918 (16)	0.0306 (5)	
C29	0.30724 (16)	0.4809 (2)	0.4545 (2)	0.0364 (6)	
C30	0.3258 (2)	0.4026 (3)	0.5276 (3)	0.0606 (10)	
H30A	0.3728	0.3735	0.5200	0.091*	
H30B	0.2891	0.3494	0.5196	0.091*	
H30C	0.3279	0.4323	0.5923	0.091*	

### Atomic displacement parameters (Å^2^)

**Table:**

	*U*^11^	*U*^22^	*U*^33^	*U*^12^	*U*^13^	*U*^23^
N5	0.094 (3)	0.100 (4)	0.139 (5)	−0.025 (3)	−0.018 (3)	0.045 (3)
C31	0.072 (3)	0.080 (3)	0.063 (3)	−0.039 (3)	0.000 (2)	0.012 (2)
C32	0.075 (3)	0.072 (3)	0.056 (2)	−0.030 (2)	0.005 (2)	0.004 (2)
C1	0.0220 (11)	0.0240 (12)	0.0226 (12)	0.0000 (9)	0.0053 (9)	0.0049 (9)
C2	0.0225 (11)	0.0237 (12)	0.0212 (11)	0.0004 (9)	0.0028 (9)	0.0000 (9)
C3	0.0218 (11)	0.0196 (11)	0.0223 (12)	−0.0035 (9)	0.0004 (9)	0.0033 (9)
C4	0.0244 (12)	0.0209 (11)	0.0284 (13)	−0.0001 (9)	0.0005 (10)	0.0035 (10)
C5	0.0306 (13)	0.0212 (12)	0.0386 (15)	0.0017 (10)	0.0050 (11)	0.0013 (11)
C6	0.0366 (14)	0.0213 (12)	0.0397 (15)	−0.0096 (11)	0.0020 (12)	−0.0012 (11)
C7	0.0254 (13)	0.0298 (14)	0.0388 (15)	−0.0088 (10)	0.0028 (11)	0.0012 (11)
C8	0.0235 (12)	0.0268 (12)	0.0290 (13)	−0.0027 (10)	0.0039 (10)	0.0030 (10)
C9	0.0222 (12)	0.0241 (13)	0.0491 (17)	0.0017 (10)	0.0028 (11)	−0.0021 (12)
C10	0.0368 (17)	0.059 (2)	0.075 (3)	0.0022 (16)	−0.0151 (17)	0.0134 (19)
C11	0.0326 (16)	0.0463 (18)	0.079 (3)	0.0003 (14)	0.0208 (16)	−0.0147 (17)
C12	0.0227 (12)	0.0334 (14)	0.0460 (16)	−0.0069 (10)	0.0104 (11)	−0.0077 (12)
C13	0.0465 (18)	0.053 (2)	0.0511 (19)	−0.0157 (15)	0.0244 (15)	−0.0143 (16)
C14	0.0292 (15)	0.0533 (19)	0.063 (2)	0.0093 (14)	0.0066 (14)	−0.0056 (17)
C15	0.0253 (12)	0.0227 (12)	0.0188 (11)	−0.0049 (9)	0.0026 (9)	−0.0016 (9)
C16	0.0210 (11)	0.0328 (13)	0.0232 (12)	−0.0038 (10)	0.0023 (9)	−0.0027 (10)
C17	0.0255 (13)	0.0437 (16)	0.0335 (15)	−0.0083 (12)	0.0027 (11)	−0.0029 (12)
C18	0.0350 (15)	0.0412 (16)	0.0402 (16)	−0.0197 (13)	0.0034 (13)	−0.0052 (13)
C19	0.0450 (17)	0.0249 (13)	0.0415 (16)	−0.0119 (12)	0.0035 (13)	−0.0029 (12)
C20	0.0345 (14)	0.0241 (12)	0.0254 (13)	−0.0051 (10)	0.0028 (10)	−0.0013 (10)
C21	0.0205 (12)	0.0336 (14)	0.0332 (14)	0.0015 (10)	0.0013 (10)	−0.0064 (11)
C22	0.0350 (15)	0.0423 (16)	0.0419 (17)	0.0061 (13)	0.0013 (13)	0.0014 (13)
C23	0.0336 (15)	0.059 (2)	0.0427 (17)	0.0101 (14)	0.0077 (13)	−0.0114 (15)
C24	0.0373 (15)	0.0211 (12)	0.0394 (15)	0.0021 (11)	−0.0006 (12)	−0.0004 (11)
C25	0.0441 (18)	0.054 (2)	0.051 (2)	0.0076 (15)	0.0073 (15)	−0.0061 (16)
C26	0.062 (2)	0.0332 (17)	0.081 (3)	0.0076 (16)	0.004 (2)	0.0178 (17)
Cl1	0.0237 (3)	0.0302 (3)	0.0383 (4)	0.0063 (2)	0.0051 (2)	0.0025 (3)
Cl2	0.0267 (3)	0.0366 (3)	0.0235 (3)	−0.0023 (2)	0.0070 (2)	−0.0041 (2)
Cr1	0.01892 (18)	0.01898 (18)	0.01855 (18)	−0.00166 (14)	0.00090 (13)	0.00088 (15)
N1	0.0169 (9)	0.0181 (9)	0.0250 (10)	−0.0013 (7)	0.0035 (8)	−0.0003 (8)
N2	0.0195 (9)	0.0176 (9)	0.0205 (10)	−0.0011 (7)	0.0032 (8)	−0.0017 (8)
N3	0.0277 (11)	0.0304 (11)	0.0227 (11)	−0.0039 (9)	−0.0009 (9)	0.0016 (9)
C27	0.0304 (13)	0.0353 (15)	0.0234 (13)	−0.0050 (11)	0.0024 (10)	0.0041 (11)
C28	0.057 (2)	0.0473 (18)	0.0369 (17)	−0.0224 (16)	0.0029 (15)	−0.0106 (14)
N4	0.0340 (12)	0.0283 (11)	0.0291 (12)	−0.0043 (9)	0.0029 (9)	0.0024 (10)
C29	0.0433 (16)	0.0306 (14)	0.0344 (15)	−0.0051 (12)	0.0018 (12)	0.0042 (12)
C30	0.078 (3)	0.046 (2)	0.054 (2)	−0.0021 (18)	−0.0039 (19)	0.0248 (17)

### Geometric parameters (Å, °)

**Table:**

N5—C31	1.115 (6)	C16—C17	1.391 (4)
C31—C32	1.441 (7)	C16—C21	1.519 (4)
C32—H32A	0.9800	C17—C18	1.376 (4)
C32—H32B	0.9800	C17—H17A	0.9500
C32—H32C	0.9800	C18—C19	1.379 (4)
C1—N1	1.331 (3)	C18—H18A	0.9500
C1—C2	1.390 (3)	C19—C20	1.397 (4)
C1—H1A	0.9500	C19—H19A	0.9500
C2—N2	1.338 (3)	C20—C24	1.512 (4)
C2—H2A	0.9500	C21—C23	1.533 (4)
C3—C8	1.409 (3)	C21—C22	1.537 (4)
C3—C4	1.411 (3)	C21—H21A	1.0000
C3—N1	1.443 (3)	C22—H22A	0.9800
C4—C5	1.384 (3)	C22—H22B	0.9800
C4—C9	1.519 (3)	C22—H22C	0.9800
C5—C6	1.392 (4)	C23—H23A	0.9800
C5—H5A	0.9500	C23—H23B	0.9800
C6—C7	1.379 (4)	C23—H23C	0.9800
C6—H6A	0.9500	C24—C26	1.528 (4)
C7—C8	1.399 (4)	C24—C25	1.534 (4)
C7—H7A	0.9500	C24—H24A	1.0000
C8—C12	1.511 (4)	C25—H25A	0.9800
C9—C10	1.529 (4)	C25—H25B	0.9800
C9—C11	1.530 (4)	C25—H25C	0.9800
C9—H9A	1.0000	C26—H26A	0.9800
C10—H10A	0.9800	C26—H26B	0.9800
C10—H10B	0.9800	C26—H26C	0.9800
C10—H10C	0.9800	Cl1—Cr1	2.3382 (7)
C11—H11A	0.9800	Cl2—Cr1	2.3029 (7)
C11—H11B	0.9800	Cr1—N2	1.9806 (19)
C11—H11C	0.9800	Cr1—N1	1.995 (2)
C12—C14	1.528 (4)	Cr1—N3	2.099 (2)
C12—C13	1.530 (4)	Cr1—N4	2.128 (2)
C12—H12A	1.0000	N3—C27	1.127 (3)
C13—H13A	0.9800	C27—C28	1.450 (4)
C13—H13B	0.9800	C28—H28A	0.9800
C13—H13C	0.9800	C28—H28B	0.9800
C14—H14A	0.9800	C28—H28C	0.9800
C14—H14B	0.9800	N4—C29	1.128 (4)
C14—H14C	0.9800	C29—C30	1.465 (4)
C15—C20	1.406 (3)	C30—H30A	0.9800
C15—C16	1.417 (3)	C30—H30B	0.9800
C15—N2	1.441 (3)	C30—H30C	0.9800
			
N5—C31—C32	179.4 (6)	C18—C19—C20	121.3 (3)
C31—C32—H32A	109.5	C18—C19—H19A	119.3
C31—C32—H32B	109.5	C20—C19—H19A	119.3
H32A—C32—H32B	109.5	C19—C20—C15	118.0 (3)
C31—C32—H32C	109.5	C19—C20—C24	120.6 (2)
H32A—C32—H32C	109.5	C15—C20—C24	121.2 (2)
H32B—C32—H32C	109.5	C16—C21—C23	112.0 (2)
N1—C1—C2	116.0 (2)	C16—C21—C22	111.0 (2)
N1—C1—H1A	122.0	C23—C21—C22	109.8 (2)
C2—C1—H1A	122.0	C16—C21—H21A	108.0
N2—C2—C1	116.5 (2)	C23—C21—H21A	108.0
N2—C2—H2A	121.7	C22—C21—H21A	108.0
C1—C2—H2A	121.8	C21—C22—H22A	109.5
C8—C3—C4	121.2 (2)	C21—C22—H22B	109.5
C8—C3—N1	120.4 (2)	H22A—C22—H22B	109.5
C4—C3—N1	118.4 (2)	C21—C22—H22C	109.5
C5—C4—C3	118.4 (2)	H22A—C22—H22C	109.5
C5—C4—C9	119.9 (2)	H22B—C22—H22C	109.5
C3—C4—C9	121.4 (2)	C21—C23—H23A	109.5
C4—C5—C6	121.1 (2)	C21—C23—H23B	109.5
C4—C5—H5A	119.4	H23A—C23—H23B	109.5
C6—C5—H5A	119.4	C21—C23—H23C	109.5
C7—C6—C5	119.9 (2)	H23A—C23—H23C	109.5
C7—C6—H6A	120.0	H23B—C23—H23C	109.5
C5—C6—H6A	120.0	C20—C24—C26	112.8 (3)
C6—C7—C8	121.3 (2)	C20—C24—C25	110.0 (2)
C6—C7—H7A	119.4	C26—C24—C25	110.2 (3)
C8—C7—H7A	119.4	C20—C24—H24A	107.9
C7—C8—C3	118.0 (2)	C26—C24—H24A	107.9
C7—C8—C12	119.1 (2)	C25—C24—H24A	107.9
C3—C8—C12	122.9 (2)	C24—C25—H25A	109.5
C4—C9—C10	109.0 (2)	C24—C25—H25B	109.5
C4—C9—C11	113.6 (2)	H25A—C25—H25B	109.5
C10—C9—C11	111.4 (3)	C24—C25—H25C	109.5
C4—C9—H9A	107.5	H25A—C25—H25C	109.5
C10—C9—H9A	107.5	H25B—C25—H25C	109.5
C11—C9—H9A	107.5	C24—C26—H26A	109.5
C9—C10—H10A	109.5	C24—C26—H26B	109.5
C9—C10—H10B	109.5	H26A—C26—H26B	109.5
H10A—C10—H10B	109.5	C24—C26—H26C	109.5
C9—C10—H10C	109.5	H26A—C26—H26C	109.5
H10A—C10—H10C	109.5	H26B—C26—H26C	109.5
H10B—C10—H10C	109.5	N2—Cr1—N1	80.59 (8)
C9—C11—H11A	109.5	N2—Cr1—N3	168.26 (8)
C9—C11—H11B	109.5	N1—Cr1—N3	87.72 (8)
H11A—C11—H11B	109.5	N2—Cr1—N4	102.18 (8)
C9—C11—H11C	109.5	N1—Cr1—N4	173.95 (8)
H11A—C11—H11C	109.5	N3—Cr1—N4	89.57 (9)
H11B—C11—H11C	109.5	N2—Cr1—Cl2	93.30 (6)
C8—C12—C14	111.3 (2)	N1—Cr1—Cl2	101.24 (6)
C8—C12—C13	111.2 (2)	N3—Cr1—Cl2	87.90 (6)
C14—C12—C13	110.5 (2)	N4—Cr1—Cl2	84.06 (7)
C8—C12—H12A	107.9	N2—Cr1—Cl1	93.62 (6)
C14—C12—H12A	107.9	N1—Cr1—Cl1	92.03 (6)
C13—C12—H12A	107.9	N3—Cr1—Cl1	87.83 (6)
C12—C13—H13A	109.5	N4—Cr1—Cl1	82.46 (7)
C12—C13—H13B	109.5	Cl2—Cr1—Cl1	165.88 (3)
H13A—C13—H13B	109.5	C1—N1—C3	117.9 (2)
C12—C13—H13C	109.5	C1—N1—Cr1	111.36 (15)
H13A—C13—H13C	109.5	C3—N1—Cr1	130.52 (15)
H13B—C13—H13C	109.5	C2—N2—C15	114.74 (19)
C12—C14—H14A	109.5	C2—N2—Cr1	111.55 (15)
C12—C14—H14B	109.5	C15—N2—Cr1	132.96 (15)
H14A—C14—H14B	109.5	C27—N3—Cr1	163.3 (2)
C12—C14—H14C	109.5	N3—C27—C28	178.6 (3)
H14A—C14—H14C	109.5	C27—C28—H28A	109.5
H14B—C14—H14C	109.5	C27—C28—H28B	109.5
C20—C15—C16	121.2 (2)	H28A—C28—H28B	109.5
C20—C15—N2	120.1 (2)	C27—C28—H28C	109.5
C16—C15—N2	118.6 (2)	H28A—C28—H28C	109.5
C17—C16—C15	117.8 (2)	H28B—C28—H28C	109.5
C17—C16—C21	119.0 (2)	C29—N4—Cr1	168.1 (2)
C15—C16—C21	123.2 (2)	N4—C29—C30	177.9 (3)
C18—C17—C16	121.6 (3)	C29—C30—H30A	109.5
C18—C17—H17A	119.2	C29—C30—H30B	109.5
C16—C17—H17A	119.2	H30A—C30—H30B	109.5
C17—C18—C19	120.1 (3)	C29—C30—H30C	109.5
C17—C18—H18A	120.0	H30A—C30—H30C	109.5
C19—C18—H18A	120.0	H30B—C30—H30C	109.5
